# Impact of renal function-based anti-tuberculosis drug dosage adjustment on efficacy and safety outcomes in pulmonary tuberculosis complicated with chronic kidney disease

**DOI:** 10.1186/s12879-019-4010-7

**Published:** 2019-05-02

**Authors:** Nayuta Saito, Yutaka Yoshii, Yugo Kaneko, Akio Nakashima, Tsugumi Horikiri, Zenya Saito, Sho Watanabe, Akira Kinoshita, Keisuke Saito, Kazuyoshi Kuwano

**Affiliations:** 1 Division of Respiratory Diseases, Department of Internal Medicine, The Jikei University Daisan Hospital, Tokyo, Japan; 2 Division of Respiratory Diseases, Department of Internal Medicine, The Jikei University School of Medicine, 3-25-8 Nishi-shimbashi, Tokyo, 105-8461 Japan; 3 Division of Nephrology and Hypertension, Department of Internal Medicine, The Jikei University Daisan Hospital, Tokyo, Japan; 4 Division of Molecular Epidemiology, The Jikei University School of Medicine, Minato-ku, Japan

**Keywords:** Chronic renal insufficiency, Drug-related side effects, Glomerular filtration rate, Hospital mortality, Pulmonary tuberculosis

## Abstract

**Background:**

Dosages of anti-tuberculosis (TB) drugs are recommended to be adjusted according to renal function for patients complicated with chronic kidney disease (CKD). However, the efficacy and safety outcomes of such renal function-based dosage adjustments are not fully elucidated.

**Methods:**

We retrospectively reviewed cases of pulmonary TB susceptible to first-line drugs that were treated at Jikei University Daisan Hospital between 2005 and 2014 with standard regimens based on dosage adjustments according to renal function recommended by international guidelines. Patients were divided into four groups, those with no, mild, moderate or severe CKD. In-hospital TB-related mortality, the rate of sputum culture conversion at 2 months, the frequency of adverse events (AEs), for which at least the temporal discontinuation of the suspect drug was required for patient improvement, and the rate of regimen change due to AEs were assessed.

**Results:**

In the 241 enrolled patients (mean age, 64.1 years; 143 men), fourteen patients (5.8%) died due to TB during their hospitalization. The rate of sputum culture conversion at 2 months was 78.0%. The frequency of in-hospital TB-related death and the conversion rate in the groups did not vary significantly according to CKD severity including those in the non-CKD group (*P* = 0.310 and *P* = 0.864). Meanwhile, a total of 70 AEs were observed in 60 patients (24.9%) and the difference between the groups in the overall frequency of AEs was almost significant (*P* = 0.051). Moreover, for the 154 patients with CKD, severe CKD stage was a significant risk factor for regimen change (OR = 5.92, 95% CI = 1.08–32.5, *P* = 0.041), as were drug-induced hepatitis and cutaneous reaction (OR = 35.6, 95% CI = 8.70–145, *P* < 0.001; OR = 17.4, 95% CI = 3.16–95.5, *P* = 0.001; respectively).

**Conclusions:**

Adjusting the dosage of TB treatment for CKD patients according to the guidelines was efficient in terms of similar therapeutic outcome to that of the non-CKD group. However, AEs warrant attention to avoid regimen change in patients with severe CKD, even if the renal function-based dosage adjustment is performed.

**Electronic supplementary material:**

The online version of this article (10.1186/s12879-019-4010-7) contains supplementary material, which is available to authorized users.

## Background

Tuberculosis (TB) continues to be a major burden in high- and low-income countries [1]. Treatment success is obviously crucial for TB management strategies because treatment failure fuels the incidence of active TB and the resistance to anti-TB drugs. Nevertheless, a poor treatment response or unsuccessful treatment sometimes occurs that can lead to an individual fatal result. Although several reasons for treatment failure exist, one of the most important issues is comorbidity, which can often present a challenge to treatment, sometimes resulting in unsuccessful treatment and death [2–4]. A strong understanding of the relationship between TB treatment and comorbidity can be of considerable clinical importance in achieving treatment success.

Chronic kidney disease (CKD) has attracted attention as a representative contributing comorbidity of TB development caused by decreased cellular immunity, vitamin D deficiency and malnutrition [5–8]. The reported overall mortality of TB patients with uraemia varies between studies, ranging from 16.4 to 36.8%, depending on the delay in diagnosis and whether therapy was discontinued due to incomplete adherence to anti-TB drugs or drug-related side effects [9–12]. Thus, administering the appropriate treatment for TB patients with CKD is of great importance in reducing mortality.

In TB patients with CKD, an inappropriate dosage of anti-TB drugs can result in unsuccessful treatment or side effects. Current guidelines for first-line anti-TB drugs therefore recommend that dosages of ethambutol (EMB) and pyrazinamide (PZA) be adjusted according to patient renal function and body weight, although no change in dosage is necessary for patients with mild renal insufficiency [13–17]. However, it remains unknown how the renal function-based dosage adjustments recommended by the guidelines affect efficacy outcomes for TB patients with CKD. Also unclear is how this affects the frequency of drug-related side effects in patients with CKD because of the differences in the corresponding dosages of anti-TB drugs for each severity of CKD between previous studies and the current guidelines. Therefore, it is critical to elucidate the impact of renal function-based dosage adjustments on frequencies of drug-related side effects and regimen change in patients with CKD of all degrees of severity. In addition, it is also imperative to investigate the impact of renal function-based dosage adjustments on measures of therapeutic efficacy, such as in-hospital mortality and sputum culture conversion rate at 2 months.

A therapeutic strategy for the administration of first-line anti-TB drugs, herein referred to as the ‘Jikei protocol’, has been applied clinically at our institution in Japan since 2004. This protocol (Additional file 1: Table S1), which brought together a combination of international and Japanese guidelines available at that time, is comparable to the current international guidelines [13–17]. We therefore consider that our clinical data for TB patients with CKD treated according to this protocol are suitable for investigating the influence of first-line anti-TB drugs on the therapeutic effects and frequency of side effects associated with these drugs. The purpose of the present study was to investigate the in-hospital TB-related mortality of pulmonary TB patients with CKD who were treated according to the recommended renal function-based dosage adjustments to standard regimen therapy, the frequency and types of drug-related side effects, and the number of patients who experienced regimen change because of side effects.

## Methods

### Study population

Hospitalized patients diagnosed as having active TB at Jikei University Daisan Hospital between July 2005 and September 2014 were retrospectively assessed for eligibility. Inclusion criteria were 1) a positive sputum culture of *Mycobacterium tuberculosis*; 2) pulmonary TB; 3) *M. tuberculosis* susceptible to all four standard drugs, isoniazid (INH), rifampicin (RMP), EMB and PZA; 4) hospitalization in compliance with the recommendation for admission and discharge of tuberculosis patients by the Ministry of Health, Labour and Welfare of Japan [18]; and 5) The adjusted dosage of each drug was based on each patient’s renal function according to the Jikei protocol (Additional file 1: Table S1). Moreover, due to drug administration issues, permitted dosage ranges of the standard drugs administered in the present study in variation of the Jikei protocol included INH: 3.5–6.5 mg/kg; RFP: 7–13 mg/kg; EB: 12.5–22.5 mg/kg (for patients without dialysis), 7.5–17.5 mg/kg (for patients with dialysis); and PZA: 17.5–32.5 mg/kg.

Exclusion criteria were as follows: A) patients having only extra-pulmonary TB, because therapeutic effects were difficult to evaluate; B) negative sputum culture; C) patients without treatment or patients in whom inadequate or excessive dosages of the standard regimen were administered; D) patients with active TB caused by *M. tuberculosis* resistant against any first-line anti-TB drugs; E) socially hospitalized patients.

### Ethics approval and consent to participate

This study was approved by the ethics committee of Jikei University School of Medicine [No. 27–031(7938)]. Informed consent was waived by the ethics committee because we retrospectively collected data without using identifying information or applying any interventions.

### Data collection

Data obtained from the patients’ medical records included age, sex, body mass index (BMI), underlying diseases, duration of hospital stay, laboratory data (including estimated glomerular filtration rate [eGFR] and human immunodeficiency virus antibody) and sputum smear grade on admission. The presence of cavitation on the admission chest X-ray was recorded. Adverse events (AEs) were evaluated and the suspect drug was recorded. The suspect drug had been determined based on clinical course and a positive result on the drug-induced lymphocyte stimulation test, which was administered as deemed necessary. The alternative drugs were also recorded if the regimen was changed. Negative conversion of the sputum culture at 2 months after starting treatment was noted, as was in-hospital TB-related death.

### Patient groups by renal insufficiency

Renal insufficiency was defined based on eGFR level according to the ‘Kidney Disease: Improving Global Outcomes’ guideline [19]. eGFR was calculated based on patient age and creatine levels using the Japanese Society of Nephrology formula [20]. Patients were divided into four groups based on their eGFR level: non-CKD, ≥90 mL/min/1.73 m^2^; mild CKD, 89–60 mL/min/1.73 m^2^; moderate CKD, 59–30 mL/min/1.73 m^2^; and severe CKD, < 30 mL/min/1.73 m^2^ or patient treatment with haemodialysis. Proteinuria was not part of the CKD definition in the present study.

### Admission and discharge criteria for active TB

The criteria for admission and discharge for active TB, which have been established by the Ministry of Health, Labour and Welfare of Japan [18], were followed in the present study. The admission criteria are a) patients with positive sputum smear or b) patients with negative sputum smear, who have a poor understanding of their disease or who are at high risk of spreading *M. tuberculosis* to others (i.e. patients with mental disorders, immunocompromised patients or those living in a group home). The criteria for discharge are a) symptoms are completely improved for more than 2 weeks after the administration of a standard regimen, (b) all results of three consecutive sputum smears or cultures are negative at more than 2 weeks after the administration of a standard regimen, and (c) the patient can be expected to adhere to anti-tuberculosis treatment after discharge with adequate knowledge about infection control of *M. tuberculosis*.

### Anti-TB treatment and dosage of each anti-TB drug

In addition to supplementation with pyridoxine, a standard four-drug regimen of INH, RMP, EMB and PZA was administered to patients aged < 80 years and a standard three-drug regimen of INH, RMP and EMB to patients ≥80 years based on the recommendation of the Japanese Society of Tuberculosis (JSTB) [16]. The ‘directly observed treatment, short course’ TB control strategy was also performed for all patients.

### Definitions of therapeutic efficacy

In-hospital TB-related death was defined as death caused by the deterioration of TB and not by other fatal diseases such as nosocomial infection. The sputum culture conversion rate at 2 months of treatment was defined as the proportion of patients under treatment with a change to a negative sputum culture.

### Definitions of adverse events, discontinuation, dosage reduction, and regimen change

AEs were defined as severe drug-related side effects that required at least temporal discontinuation of the suspect drugs causing the AEs. When an anti-TB drug caused side effect(s), physicians discontinued the drug temporarily based on the severity of the reaction. This temporal interruption of anti-TB drug(s) was defined as discontinuation. Desensitisation therapy for the drug was administered after the improvement of AEs in accordance with JSTB protocol [21]. When a patient was re-administered the initial drug, but at a decreased dose following desensitisation therapy, this dosage decrease was defined as dosage reduction. When AEs recurred during desensitisation therapy, the suspect drug was permanently discontinued, and the regimen was converted with or without alternative drug(s). This conversion due to the AEs was defined as regimen change.

### Definitions of individual adverse events

Drug-induced hepatitis is the elevation of serum aspartate transaminase/alanine transaminase to more than five times the upper limit of normal or the elevation of total bilirubin levels to 2.0 mg/dL or more, with or without symptoms. Cutaneous reaction is a rash with blisters or necrosis covering more than one-third of the body surface. Gastrointestinal disorder is the presence of gastrointestinal symptoms such as nausea, abdominal pain, diarrhoea and anorexia. Drug-induced nephropathy is the presence of elevated serum creatinine to > 2.0 mg/dL in response to drug administration or interstitial nephritis revealed by renal biopsy. Gout attack is an acute arthritis with hyperuricaemia. Haematotoxicity includes anaemia (haemoglobin levels <7.0 g/dL), leukopaenia (<2000/μL), neutropaenia (<1000/μL) and/or thrombocytopaenia (<100,000/μL). Peripheral neuropathy is the presence of neurological symptoms of impaired sensation, movement and gland function in the hands and/or feet. Anaphylaxis is a serious allergic reaction involving the skin or mucosal tissue with shock.

### Statistical analysis

The chi-square test was used to compare each proportion. One-way ANOVA was used to compare differences in mean values. Variables with *P* < 0.05 in the univariate analysis and sex were included in multivariate logistic regression analyses in a stepwise selection process. Odds ratios (OR) were calculated with 95% confidence intervals (CI). A *P* value <0.05 indicated statistical significance in all analyses. Statistical analyses were performed using Graph Pad Prism 7 (GraphPad Software, La Jolla, CA, USA) or STATA 14.0 (STATA Corp., College Station, TX, USA).

## Results

### Patient characteristics

**Fig. 1:**
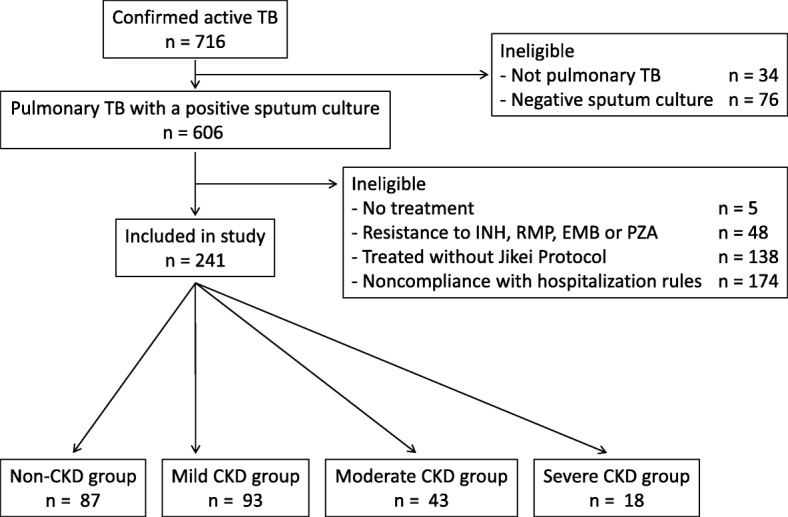
Eligibility flow chart for the study subjects. CKD: chronic kidney disease; EMB: ethambutol; INH: isoniazid; PZA: pyrazinamide; RMP: rifampicin; TB: tuberculosis

During the study period, 716 patients were diagnosed as having active TB, of whom 241 patients were enrolled in the present study (Fig. 1): 87 (36.1%) in the non-CKD group, 93 (38.6%) in the mild CKD group, 43 (17.8%) in the moderate CKD group and 18 (7.5%) in the severe CKD group (including 11 patients on dialysis) (Table 1). The overall mean age was 64.1 years, and 143 patients (59.3%) were men. The underlying diseases in each group are summarised in Table 1. No patient was infected with the human immunodeficiency virus. Of the included patients, 164 (68.0%) were treated with the INH, RMP, EMB and PZA (HREZ) regimen and 77 (32.0%) with the INH, RMP and EMB (HRE) regimen. The number of patients in the severe CKD group treated with HREZ was lower than the number treated with HRE (5 patients; 27.8% vs 13 patients; 72.2%), most likely due to a greater number of elderly patients in the severe CKD group. There were no significant differences between the four groups in mean dosage per kilogram of drugs, except for the adjusted reduction of EMB, reflecting the protocol. However, sputum smear-positive and cavitation rates were significantly different between the four groups (*P* < 0.001, respectively).

**Table 1:** Patient characteristics

	Total	Non-CKD	CKD group			Subtotal of CKD groups	*P* value*
			Mild CKD	Moderate CKD	Severe CKD		
	*n* = 241	*n* = 87	*n* = 93	*n* = 43	*n* = 18	*n* = 154	
Age, y (mean ± SD)	64.1 ± 21.7	50.2 ± 20.8	65.5 ± 19.2	82.2 ± 9.6	81.2 ± 10.2	72.0 ± 18.0	<0.001
Male, n (%)	143 (59.3%)	53 (60.9%)	58 (62.4%)	21 (48.8%)	11 (61.1%)	90 (58.4%)	0.488
BMI, kg/m^2^ (mean ± SD)	19.0 ± 2.4	19.1 ± 2.0	19.1 ± 2.6	18.9 ± 2.7	18.3 ± 2.9	19.0 ± 2.6	0.517
Body weight, kg (range)	27.8–64.4	30.8–60.0	27.8–60.0	30.0–60.0	31.5–64.4	27.8–64.4	–
Current or ex-smoker, n (%)	99 (41.1%)	38 (43.7%)	41 (44.1%)	13 (30.2%)	7 (38.9%)	61 (39.6%)	0.438
Past history of TB treatment, n (%)	69 (28.6%)	14 (16.1%)	31 (33.3%)	20 (46.5%)	4 (22.2%)	55 (35.7%)	0.002
Underlying disease							
Diabetes mellitus	77 (32.0%)	30 (34.5%)	28 (30.1%)	14 (32.6%)	5 (27.8%)	47 (30.5%)	0.907
Pulmonary disease	26 (10.8%)	7 (8.0%)	10 (10.8%)	4 (9.3%)	5 (27.8%)	19 (12.3%)	0.103
Malignancy	20 (8.3%)	7 (8.0%)	7 (7.5%)	5 (11.6%)	1 (5.6%)	13 (8.4%)	0.829
Liver damage	18 (7.5%)	6 (6.9%)	9 (9.7%)	2 (4.7%)	1 (5.6%)	12 (7.8%)	0.732
Alcoholic liver disease	3 (1.2%)	1 (1.1%)	2 (2.2%)	0 (0%)	0 (0%)	2 (1.3%)	–
Hepatitis C virus	3 (1.2%)	0 (0%)	1 (1.1%)	2 (4.7%)	0 (0%)	3 (1.9%)	–
Autoimmune hepatitis	2 (0.8%)	0 (0%)	2 (2.2%)	0 (0%)	0 (0%)	2 (1.3%)	–
Hepatitis B virus	1 (0.4%)	0 (0%)	1 (1.1%)	0 (0%)	0 (0%)	1 (0.6%)	–
Others	9 (3.7%)	5 (5.7%)	3 (3.2%)	0 (0%)	1 (5.6%)	4 (2.6%)	–
Immunosuppressive drug use, n (%)	33 (13.7%)	8 (9.2%)	11 (11.8%)	12 (28.0%)	2 (11.1%)	25 (16.2%)	0.027
Sputum smear positive, n (%)	210 (87.1%)	78 (89.7%)	82 (88.2%)	41 (95.3%)	9 (50.0%)	132 (85.7%)	<0.001
Cavitation on chest X-ray, n (%)	105 (43.6%)	54 (62.1%)	36 (38.7%)	11 (25.6%)	4 (22.2%)	51 (33.1%)	<0.001
Laboratory data							
WBC count, cells/mL (mean ± SD)	7258 ± 2789	7492 ± 2440	7206 ± 2798	7121 ± 2612	6722 ± 4455	7126 ± 2968	0.705
Lymphocyte, cells/mL (mean ± SD)	1054 ± 619	1180 ± 654	1120 ± 609	819 ± 485	667 ± 495	983 ± 588	<0.001
Albumin, g/dL (mean ± SD)	3.2 ± 0.8	3.4 ± 0.8	3.4 ± 0.7	2.9 ± 0.7	2.4 ± 0.9	3.1 ± 0.8	<0.001
Treatment regimen, n (%)							
HREZ	164 (68.0%)	75 (86.2%)	69 (74.2%)	15 (34.9%)	5 (27.8%)	89 (57.8%)	< 0.001
HRE	77 (32.0%)	12 (13.8%)	24 (25.8%)	28 (65.1%)	13 (72.2%)	65 (42.2%)	–
Treatment dosage, mg/kg (mean ± SD)							
INH	5.6 ± 0.5	5.5 ± 0.5	5.6 ± 0.5	5.6 ± 0.5	5.4 ± 0.5	5.6 ± 0.5	0.689
RMP	8.9 ± 1.2	8.8 ± 1.1	8.8 ± 1.1	9.3 ± 1.4	9.0 ± 1.4	9.0 ± 1.2	0.089
EMB	14.3 ± 1.8	14.3 ± 1.6	14.6 ± 1.7	14.6 ± 1.6	12.6 ± 2.9	14.3 ± 2.0	<0.001
PZA	23.3 ± 2.3	23.2 ± 2.1	23.3 ± 2.1	24.0 ± 3.0	23.5 ± 4.5	23.4 ± 2.4	0.692

*One-way ANOVA or Chi-square test was used to compare for each parameter

*BMI* body mass index, *CKD* chronic kidney disease, *EMB* ethambutol, *HRE* isoniazid: rifampicin and ethambutol, *HREZ* isoniazid, rifampicin, ethambutol and pyrazinamide, *INH* isoniazid; min, minimum; max, maximum, *PZA* pyrazinamide, *RMP* rifampicin, *SD* standard deviation, *TB* tuberculosis, *WBC* white blood cell

### Impact of renal function-based dosage adjustment on adverse events due to anti-TB treatment and therapeutic efficacy in patients with CKD

In total, 70 AEs were observed in 60 patients (Table 2). The most frequent AEs were drug-induced hepatitis in 28 patients (11.6% of all patients) followed by cutaneous reaction in 19 patients (7.9%). Suspect drugs causing AEs are listed in Additional file 2: Table S2. The drug most frequently associated with AEs was PZA in 21 patients (30.0%), followed by RFP in 10 patients (14.3%); meanwhile, the suspect drug causing AEs was unable to be definitely determined in 26 patients (37.1%). There was no significant difference between the groups in the overall number of AEs (*P =* 0.051), although the overall frequencies of AEs in moderate and severe CKD were relatively higher than the rate in the non-CKD group (34.9, 44.4 and 20.7%, respectively). For each AE, there were no differences between the groups in the frequencies of drug-induced hepatitis or cutaneous reaction (*P* = 0.252 and *P* = 0.855, respectively).

**Table 2:** Therapeutic effects, adverse events and regimen changes in the four patient groups

	Total	Non-CKD	CKD group			Subtotal of CKD groups	*P* value^*^
			Mild CKD	Moderate CKD	Severe CKD		
	*n* = 241	*n* = 87	*n* = 93	*n* = 43	*n* = 18	*n* = 154	
Therapeutic effects							
In-hospital TB-related death	14 (5.8%)	3 (3.4%)	5 (5.4%)	5 (11.6%)	1 (5.6%)	11 (7.1%)	0.310
Sputum culture conversion at 2 months^†^	174 (78.0%)	67 (77.9%)	66 (76.7%)	28 (77.8%)	13 (86.7%)	107 (78.1%)	0.864
Any adverse events	60 (24.9%)	18 (20.7%)	19 (20.4%)	15 (34.9%)	8 (44.4%)	42 (27.3%)	0.051
No adverse event	181 (75.1%)	69 (79.3%)	74 (79.6%)	28 (65.1%)	10 (55.6%)	112 (72.7%)	–
Drug-induced hepatitis	28 (11.6%)	6 (6.9%)	12 (12.9%)	8 (18.6%)	2 (11.1%)	22 (14.3%)	0.252
Cutaneous reaction	19 (7.9%)	8 (9.2%)	6 (6.5%)	3 (7.0%)	2 (11.1%)	11 (7.1%)	0.855
Drug-induced nephropathy	7 (2.9%)	0 (0%)	0 (0%)	5 (11.6%)	2 (11.1%)	7 (4.5%)	N/A^‡^
Gastrointestinal disorder	6 (2.5%)	1 (1.1%)	2 (2.2%)	1 (2.3%)	2 (11.1%)	5 (3.2%)	N/A
Gout attack	5 (2.1%)	4 (4.6%)	0 (0%)	0 (0%)	1 (5.6%)	1 (0.6%)	N/A
Haematotoxicity	3 (1.2%)	0 (0%)	2 (2.2%)	0 (0%)	1 (5.6%)	3 (1.9%)	N/A
Peripheral neuropathy	1 (0.4%)	1 (1.1%)	0 (0%)	0 (0%)	0 (0%)	0 (0%)	N/A
Anaphylaxis	1 (0.4%)	0 (0%)	0 (0%)	1 (2.3%)	0 (0%)	1 (0.6%)	N/A
Regimen change	28 (11.6%)	3 (3.4%)	10 (10.8%)	9 (20.9%)	6 (33.3%)	25 (16.2%)	< 0.001

There was some overlap

*Chi-square test was used to compare for each parameter

^‡^N/A, chi-square test was not applicable because of insufficient expected values in some cells

^§^The number of patients alive at 2 months was 223 (86 patients in the non-CKD group, 86 in the mild CKD group, 36 in the moderate CKD group and 15 in the severe CKD group)

*CKD* chronic kidney disease

Meanwhile, fourteen patients (5.8%) died due to TB during hospitalisation and the sputum culture conversion rate at 2 months was 78% across all patients. (Table 2). There was no significant difference between the groups with regard to in-hospital TB-related mortality and sputum culture conversion rate (*P* = 0.310 and 0.864, respectively).

### Relationship between frequency of regimen change and severity of CKD

After development of AEs, desensitisation therapy enabled 27 patients to resume the same dosage of the drug as initially administered prior to discontinuation. Dosage reduction was required in 5 patients (Additional file 3: Table S3). Regimen change was required for 28 patients (11.6% of all patients; 46.7% of the 60 patients who experienced AEs; Table 2 and Additional file 3: Table S3); There was a significant difference in the frequency of regimen change between the groups (*P* < 0.001;). Multivariate logistic analysis in CKD patients revealed that severe CKD, drug-induced hepatitis and cutaneous reaction were significant risk factors for regimen change (OR = 5.92, 95% CI = 1.08–32.5, *P* = 0.041; OR = 35.6, 95% CI = 8.70–145, *P* < 0.001; and OR = 17.4, 95% CI = 3.16–95.5, *P* = 0.001; respectively; Table 3). The alternative drugs used are listed in Additional file 3: Table S3; levofloxacin was the most frequently administered drug at 42.9% of the 28 patients who required regimen change.

**Table 3:** Univariate and multivariate analyses of risk factors for regimen change in the CKD patients

	Regimen change		Univariate			Multivariate		
	Yes	No	Odds ratio	95% CI	*P* value	Odds ratio	95% CI	*P* value
	*n* = 25	*n* = 129						
Male, n (%)	12 (48.0%)	78 (60.5%)	0.60	0.26–1.43	0.250	1.94	0.55–6.85	0.305
BMI, kg/m^2^ (mean ± SD)	19.1 ± 2.60	19.0 ± 2.66	1.01	0.86–1.19	0.858			
CKD severity								
Mild	10 (40.0%)	83 (64.3%)	Reference	–	–	Reference	–	–
Moderate	9 (36.0%)	34 (26.4%)	2.20	0.82–5.88	0.117	2.10	0.57–7.71	0.264
Severe	6 (24.0%)	12 (9.3%)	4.15	1.28–13.5	0.018	5.92	1.08–32.5	0.041
Underlying disease								
Liver damage	2 (8.0%)	10 (7.8%)	1.03	0.21–5.04	0.966			
Laboratory data								
Albumin, g/dL (mean ± SD)	2.6 ± 0.8	3.2 ± 0.8	0.41	0.23–0.73	0.003	0.68	0.31–1.46	0.320
Treatment regimen, n (%)								
HREZ	13 (52.0%)	76 (58.9%)	0.755	0.32–1.78	0.523			
Adverse events								
Drug-induced hepatitis, n (%)	14 (56.0%)	8 (6.2%)	19.3	6.63–55.9	<0.001	35.6	8.70–145	<0.001
Cutaneous reaction, n (%)	6 (24.0%)	5 (3.9%)	7.83	2.17–28.2	0.002	17.4	3.16–95.5	0.001

*BMI* body mass index, *CI* confidence interval, *CKD* chronic kidney disease, *HREZ* isoniazid, rifampicin, ethambutol and pyrazinamide, *SD* standard deviation

## Discussion

Reducing TB mortality is one of the principal targets of TB management [22]. Several previous studies have reported in-hospital mortality rates resulting from active TB ranging from 1.8–17.3% [23–28]. The 5.8% in-hospital TB-related mortality rate in the present study was comparable to previous reports. The frequency of in-hospital TB-related death did not vary significantly with the presence of CKD or CKD severity (3.4–11.6%). In addition, conversion rate was not significantly different between the groups (76.7–86.7%), although the conversion rate results might have been biased by high smear-positive and cavitation rates in the non-CKD group. These results show that renal function-based dosage adjustments for CKD patients recommended by the current guidelines result in favourable outcomes in terms of similar therapeutic efficacy to non-CKD patients. Hence, administration of therapy according to the renal function-based dosage adjustment may reduce TB mortality in patients with CKD.

Anti-TB drugs can cause various drug-related side effects ranging from mild to life threatening [13]. In the present study, drug-induced hepatitis (11.6%) was the leading AE followed by cutaneous reaction (7.9%). The frequency of drug-induced hepatitis was consistent with previous reports (2.3–16.1%) [29–32]. There were no significant differences in the frequency of the two major AEs, drug-induced hepatitis and cutaneous reaction in the present study (*P* = 0.252 and *P* = 0.855, respectively). However, the result for the overall frequency of AEs between the groups was extremely close to being statistically significant (*P* = 0.051) and showed an increasing frequency according to CKD severity (20.4–44.4%). This result suggests that the CKD patients tended to develop AEs compared to non-CKD patients, even though renal function-based dosage adjustment was performed. Moreover, the high rate of AEs might have resulted from high mean age, since advanced age is associated with increased side effects from first-line anti-TB drugs [33]. Thus, closer observation is needed in CKD patients to ensure the earlier detection of side effects.

Drug-related side effects sometimes require the permanent discontinuation of an initial drug and thus a change of regimen to an alternative second-line drug such as fluoroquinolone. However, as regimens containing fluoroquinolone result in poorer outcomes and more frequent side effects [34], regimen changes should be strongly avoided. In the present study, severe CKD was a significant risk factor for regimen change in the CKD patients, with respective rates of 33.3% in the severe CKD group versus 3.4% in the non-CKD group, as were drug-induced hepatitis and cutaneous reactions. A pharmacokinetic study revealed the significant accumulation of INH in CKD patients because of reduced hepatic acetylation compared to normal subjects [35]. Hence, the accumulation of INH may be prolonged in CKD patients even after improvements in symptoms and laboratory findings. If a severe drug-related side effect occurs in a CKD patient during a standard treatment regimen, a longer discontinuation period for the regimen may improve success of desensitisation therapy, thus preventing resistance to second-line drugs.

The present study has some limitations. First, this is a retrospective study at a single centre, although one strength is the large number of CKD patients included. Second, for comparison with the analysed groups, there was no control group in which no anti-TB drug dosage adjustment based on renal function was performed. However, it is ethically unacceptable to administer dosages outside the permissible range recommended by the guidelines. Third, more than half of the enrolled patients were excluded from the present study because of ineligibility based on factors such as social hospitalisation or treatment outside the permissible dosage range. In addition, the definitions of AEs used in this study are stricter than typically used in the clinical setting. Hence, there is a possibility that mild side effects may not have been detected. Fourth, the EMB dosage for patients undergoing haemodialysis in the Jikei protocol was slightly low compared to international guidelines. Moreover, the HREZ regimen was used in fewer patients than the HRE regimen in the severe CKD group. These differences might affect safety and efficacy. Finally, it was not possible to identify which drug had a specific role in the development of drug-related side effects in some cases. Thus, a larger-scale prospective study that takes blood concentrations of the drugs into account is therefore required.

## Conclusions

The present findings clarified that renal function-based dosage adjustments of anti-TB drugs based on the current guidelines can be applied effectively in CKD patients without increasing in-hospital TB-related mortality. Physicians should be aware of the increased risk of regimen change in severe CKD patients and therefore ensure that drug-related side effects are addressed effectively if they occur.

## Additional files

Additional file1: **Table S1**. Jikei protocol and dosages of first-line anti-tuberculosis drugs for each level of renal insufficiency (DOCX 19 kb)

Additional file2: **Table S2**. Suspect drugs for each adverse event (DOCX 19 kb)

Additional file3: **Table S3**. Details of treatment after desensitisation therapy (DOCX 15 kb)

## Abbreviations

AE: adverse event
BMI: body mass index
CCr: creatinine clearance
CI: confidence interval
CKD: chronic kidney disease
eGFR: estimated glomerular filtration rate
EMB: ethambutol
HD: haemodialysis
INH: isoniazid
JSTB: The Japanese Society for tuberculosis
OR: odds ratio
PZA: pyrazinamide
RMP: rifampicin
TB: tuberculosis
