# Supplementary material for: Impact of renal function-based anti-tuberculosis drug dosage adjustment on efficacy and safety outcomes in pulmonary tuberculosis complicated with chronic kidney disease
Source: BMC Infect Dis. 2019 May 2;19:374. doi: 10.1186/s12879-019-4010-7 (PMC6498605; doi:10.1186/s12879-019-4010-7)
Supplement: Supplementary file 1 — Table S1. Jikei protocol and dosages of first-line anti-tuberculosis drugs for each level of renal insufficiency (DOCX 19 kb) [file 12879_2019_4010_MOESM1_ESM.docx]

**Table S1.** Jikei protocol and dosages of first-line anti-tuberculosis drugs for each level of renal insufficiency^a^

|  |  | **EMB** | | | | |  | **PZA** | | | | |
| --- | --- | --- | --- | --- | --- | --- | --- | --- | --- | --- | --- | --- |
|  |  | Jikei | WHO | ATS/CDC/IDSA | ERS | JSTB |  | Jikei | WHO | ATS/CDC/IDSA | ERS | JSTB |
| CCr≥60 mL/min | Dosage | 15 mg/kg | 15 (15–20) mg/kg | 15–25 mg/kg | 15 mg/kg | 1000 mg/body |  | 25 mg/kg | 25 (20–30) mg/kg | 25–35 mg/kg | <50 kg: 1500 mg/body or ≥50 kg: 2000 mg/body | 1500 mg/body |
|  | Frequency | q24h | q24h | q24h | q24h | q24h |  | q24h | q24h | q24h | q24h | q24h |
| 59>CCr≥30 mL/min | Dosage | 15 mg/kg | 15 (15–20) mg/kg | 15–25 mg/kg | 15 mg/kg | Dose reduction |  | 25 mg/kg | 25 (20–30) mg/kg | 25–35 mg/kg | <50 kg: 1500 mg/body or ≥50 kg: 2000 mg/body | Dose reduction |
|  | Frequency | q24h | q24h | q24h | q24h | q24h |  | q24h | q24h | q24h | q24h | q24h |
| 30>CCr mL/min | Dosage | 15 mg/kg | 15 mg/kg | 20–25 mg/kg | 15–25 mg/kg | 1000 mg/body |  | 25 mg/kg | 25 mg/kg | 25–35 mg/kg | 25–30 mg/kg | 1500 mg/body |
|  | Frequency | q48h | 3 times a week | 3 times a week | 3 times a week | q48h or 3 times a week |  | q48h | 3 times a week | 3 times a week | 3 times a week | q48h or 3 times a week |
| HD | Dosage | 10 mg/kg | 15 mg/kg | 20–25 mg/kg | 15–25 mg/kg | 750 mg/body |  | 25 mg/kg | 25 mg/kg | 25–35 mg/kg | 25–30 mg/kg | 1500 mg/body |
|  | Frequency | q48h | 3 times a week | 3 times a week | 3 times a week | After HD^b^ or 10 mg/kg q48h^c^ |  | q48h | 3 times a week | 3 times a week | 3 times a week | After HD |
|  |  |  |  |  |  |  |  |  |  |  |  |  |
|  |  | **INH** | | | | |  | **RMP** | | | | |
|  |  | Jikei | WHO | ATS/CDC/IDSA | ERS | JSTB |  | Jikei | WHO | ATS/CDC/IDSA | ERS | JSTB |
| CCr≥60 mL/min | Dosage | 5 mg/kg | 5 (4–6) mg/kg | 300 mg/body | 300 mg/body | 300 mg/body |  | 10 mg/kg | 10 (8–12) mg/kg | 600 mg/body | <50 kg: 450 mg/body or ≥50 kg: 600 mg/body | 600 mg/body |
|  | Frequency | q24h | q24h | q24h | q24h | q24h |  | q24h | q24h | q24h | q24h | q24h |
| 59>CCr≥30 mL/min | Dosage | 5 mg/kg | 5 (4–6) mg/kg | 300 mg/body | 300 mg/body | 300 mg/body |  | 10 mg/kg | 10 (8–12) mg/kg | 600 mg/body | <50 kg: 450 mg/body or ≥50 kg: 600 mg/body | 600 mg/body |
|  | Frequency | q24h | q24h | q24h | q24h | q24h |  | q24h | q24h | q24h | q24h | q24h |
| 30>CCr mL/min | Dosage | 5 mg/kg | 5 (4–6) mg/kg | 300 mg/body daily or 900 mg/body 3 times a week | 300 mg/body daily or 900 mg/body 3 times a week | 300 mg/body |  | 10 mg/kg | 10 (8–12) mg/kg | 600 mg/body daily or 600 mg/body 3 times a week | <50 kg: 450 mg/body or ≥50 kg: 600 mg/body | 600 mg/body |
|  | Frequency | q24h | q24h |  |  | q24h |  | q24h | q24h |  | q24h | q24h |
| HD | Dosage | 5 mg/kg | 5 (4–6) mg/kg | 300 mg/body daily or 900 mg/body 3 times a week | 300 mg/body daily or 900 mg/body 3 times a week | 300 mg/body |  | 10 mg/kg | 10 (8–12) mg/kg | 600 mg/body daily or 600 mg/body 3 times a week | <50 kg: 450 mg/body or ≥50 kg: 600 mg/body | 600 mg/body |
|  | Frequency | q24h | q24h |  |  | q24h |  | q24h | q24h |  | q24h | q24h |

^a^ As recommended by WHO [13], ATS/CDC/IDSA [14], ERS [15] and JSTB [16, 17]. ^b^As recommended by JSTB [16]. ^c^As recommended by JSTB [17].

ATS: American Thoracic Society; CCr: creatinine clearance; CDC: Centers for Disease Control and Prevention; EMB: ethambutol; ERS: European Respiratory Society; HD: haemodialysis; IDSA: Infectious Diseases Society of America; INH: isoniazid; JSTB: Japanese Society for Tuberculosis; PZA: pyrazinamide; RMP: rifampicin; WHO: World Health Organization
