# Supplementary material for: Impact of renal function-based anti-tuberculosis drug dosage adjustment on efficacy and safety outcomes in pulmonary tuberculosis complicated with chronic kidney disease
Source: BMC Infect Dis. 2019 May 2;19:374. doi: 10.1186/s12879-019-4010-7 (PMC6498605; doi:10.1186/s12879-019-4010-7)
Supplement: Supplementary file 2 — Table S2. Suspect drugs for each adverse event (DOCX 19 kb) [file 12879_2019_4010_MOESM2_ESM.docx]

**Table S2.** Suspect drugs for each adverse event

| Adverse event | Group | Total |  |  | Suspected drug |  |  |
| --- | --- | --- | --- | --- | --- | --- | --- |
|  |  |  | INH | RFP | EMB | PZA | Undetermined |
| Any adverse events (n = 70) |  |  |  |  |  |  |  |
|  | non-CKD | 17 (24.3%) | 0 (0%) | 4 (5.7%) | 2 (2.9%) | 5 (7.1%) | 6 (8.6%) |
|  | CKD | 53 (75.7%) | 7 (10.0%) | 6 (8.6%) | 4 (5.7%) | 16 (22.9%) | 20 (28.6%) |
|  | Total | 70 (100%) | 7 (10.0%) | 10 (14.3%) | 6 (8.6%) | 21 (30.0%) | 26 (37.1%) |
|  |  |  |  |  |  |  |  |
| Drug-induced hepatitis (n = 28) |  |  |  |  |  |  |  |
|  | non-CKD | 6 (21.4%) | 0 (0%) | 2 (7.1%) | 0 (0%) | 0 (0%) | 4 (14.3%) |
|  | CKD | 22 (78.6%) | 4 (14.3%) | 2 (7.1%) | 0 (0%) | 5 (17.9%) | 11 (39.3%) |
|  | Total | 28 (100%) | 4 (14.3%) | 4 (14.3%) | 0 (0%) | 5 (17.9%) | 15 (53.6%) |
|  |  |  |  |  |  |  |  |
| Cutaneous reaction (n = 19) |  |  |  |  |  |  |  |
|  | non-CKD | 8 (42.1%) | 0 (0%) | 1 (5.3%) | 1 (5.3%) | 4 (21.1%) | 2 (10.5%) |
|  | CKD | 11 (57.9%) | 1 (5.3%) | 1 (5.3%) | 2 (10.5%) | 4 (21.1%) | 3 (15.8%) |
|  | Total | 19 (100%) | 1 (5.3%) | 2 (10.5%) | 3 (15.8%) | 8 (42.1%) | 5 (26.3%) |
|  |  |  |  |  |  |  |  |
| Drug-induced nephropathy (n = 7) |  |  |  |  |  |  |  |
|  | non-CKD | 0 (0%) | 0 (0%) | 0 (0%) | 0 (0%) | 0 (0%) | 0 (0%) |
|  | CKD | 7 (100%) | 1 (14.3%) | 2 (28.6%) | 1 (14.3%) | 2 (28.6%) | 1 (14.3%) |
|  | Total | 7 (100%) | 1 (14.3%) | 2 (28.6%) | 1 (14.3%) | 2 (28.6%) | 1 (14.3%) |
|  |  |  |  |  |  |  |  |
| Gastrointestinal disorder (n = 6) |  |  |  |  |  |  |  |
|  | non-CKD | 1 (16.7%) | 0 (0%) | 1 (16.7%) | 0 (0%) | 0 (0%) | 0 (0%) |
|  | CKD | 5 (83.3%) | 1 (16.7%) | 0 (0%) | 0 (0%) | 1 (16.7%) | 3 (50.0%) |
|  | Total | 6 (100%) | 1 (16.7%) | 1 (16.7%) | 0 (0%) | 1 (16.7%) | 3 (50.0%) |
|  |  |  |  |  |  |  |  |
| Gout attack (n = 5) |  |  |  |  |  |  |  |
|  | non-CKD | 1 (20.0%) | 0 (0%) | 0 (0%) | 0 (0%) | 1 (20.0%) | 0 (0%) |
|  | CKD | 4 (80.0%) | 0 (0%) | 0 (0%) | 0 (0%) | 4 (80.0%) | 0 (0%) |
|  | Total | 5 (100%) | 0 (0%) | 0 (0%) | 0 (0%) | 5 (100%) | 0 (0%) |
|  |  |  |  |  |  |  |  |
| Haematotoxicity (n = 3) |  |  |  |  |  |  |  |
|  | non-CKD | 0 (0%) | 0 (0%) | 0 (0%) | 0 (0%) | 0 (0%) | 0 (0%) |
|  | CKD | 3 (100%) | 0 (0%) | 1 (33.3%) | 0 (0%) | 0 (0%) | 2 (66.7%) |
|  | Total | 3 (100%) | 0 (0%) | 1 (33.3%) | 0 (0%) | 0 (0%) | 2 (66.7%) |
|  |  |  |  |  |  |  |  |
| Peripheral neuropathy (n = 1) |  |  |  |  |  |  |  |
|  | non-CKD | 1 (100%) | 0 (0%) | 0 (0%) | 1 (100%) | 0 (0%) | 0 (0%) |
|  | CKD | 0 (0%) | 0 (0%) | 0 (0%) | 0 (0%) | 0 (0%) | 0 (0%) |
|  | Total | 1 (100%) | 0 (0%) | 0 (0%) | 1 (100%) | 0 (0%) | 0 (0%) |
|  |  |  |  |  |  |  |  |
| Anaphylaxis (n = 1) |  |  |  |  |  |  |  |
|  | non-CKD | 0 (0%) | 0 (0%) | 0 (0%) | 0 (0%) | 0 (0%) | 0 (0%) |
|  | CKD | 1 (100%) | 0 (0%) | 0 (0%) | 1 (100%) | 0 (0%) | 0 (0%) |
|  | Total | 1 (100%) | 0 (0%) | 0 (0%) | 1 (100%) | 0 (0%) | 0 (0%) |

EMB: ethambutol; INH: isoniazid; PZA: pyrazinamide; RMP: rifampicin
