# Supplementary material for: Impact of renal function-based anti-tuberculosis drug dosage adjustment on efficacy and safety outcomes in pulmonary tuberculosis complicated with chronic kidney disease
Source: BMC Infect Dis. 2019 May 2;19:374. doi: 10.1186/s12879-019-4010-7 (PMC6498605; doi:10.1186/s12879-019-4010-7)
Supplement: Supplementary file 3 — Table S3. Details of treatment after desensitisation therapy (DOCX 15 kb) [file 12879_2019_4010_MOESM3_ESM.docx]

**Table S3.** Details of treatment after desensitisation therapy

| HREZ regimen |  |  |  | |  |  |
| --- | --- | --- | --- | --- | --- | --- |
|  | Total (n = 38) | | | | non-CKD (n = 15) | CKD (n= 23) |
| Same dosage as the initial treatment | 20 (52.6%) | | | | 12 (80.0%) | 8 (34.8%) |
| Dosage reduction | 2 (5.3%) | | | | 0 (0%) | 2 (8.7%)^a^ |
| Regimen change: change to | 16 (42.1%) | | | | 3 (20.0%) | 13 (56.5%) |
| HRE | 9 (23.7%) | | | | 1 (6.7%) | 8 (34.8%) |
| HEZL | 4 (10.5%) | | | | 2 (13.3%) | 2 (8.7%) |
| Others | 3 (7.9%) | | | | 0 (0%) | 3 (13.0%) |
|  |  | | | |  |  |
| HRE regimen |  | | | |  |  |
|  | Total (n = 22) | | | | Non-CKD (n = 3) | CKD (n = 19) |
| Same dosage as the initial treatment | 7 (31.8%) | | | | 2 (66.7%) | 5 (26.3%) |
| Dosage reduction | 3 (13.6%) | | | | 1 (33.3%)^b^ | 2 (10.5%)^c^ |
| Regimen change: change to | 12 (54.5%) | | | | 0 (0%) | 12 (63.2%) |
| HRL | 4 (18.2%) | | | | 0 (0%) | 4 (21.1%) |
| HEL | 2 (9.1%) | | | | 0 (0%) | 2 (10.5%) |
| Others | 6 (27.3%) | | | | 0 (0%) | 6 (31.2%) |
|  |  |  |  |  |  |  |

^a^ 70 and 80% of the initial dosage of EMB and PZA, respectively, were re-administered to each one patient. ^b^ 70% of the initial dosages of INH, RFP, and EMB were re-administered to one patient. ^c^ 70% of the initial dosages of both RFP and EMB were re-administered to one patient. Moreover, 50% of the initial dosage of EMB was re-administered to another patient.

HEL: isoniazid, ethambutol, levofloxacin; HEZL: isoniazid, ethambutol, pyrazinamide, levofloxacin; HRE: isoniazid, rifampicin, ethambutol; HREZ: isoniazid, rifampicin, ethambutol, pyrazinamide: HRL; isoniazid, rifampicin, levofloxacin: HRZS; isoniazid, rifampicin, pyrazinamide, streptomycin
